# Structure prediction, docking studies and molecular cloning of novel *Pichia kudriavzevii* YK46 *metalloprotease* (*MetPr*) for improvement of feather waste biodegradation

**DOI:** 10.1038/s41598-023-47179-5

**Published:** 2023-11-15

**Authors:** Nagwa M. Abd El-Aziz, Bigad E. Khalil, Nora N. El-Gamal

**Affiliations:** 1 Microbial Genetic Department, Biotechnology Research Institute, National Research Centre, 33 El Buhouth ST, Dokki, Cairo, 12622 Egypt; 2 Microbial Chemistry Department, Biotechnology Research Institute, National Research Centre, 33 El Buhouth ST, Dokki, Cairo, 12622 Egypt

**Keywords:** Biotechnology, Genetics, Microbiology

## Abstract

This study addresses the environmental risks associated with the accumulation of keratin waste from poultry, which is resistant to conventional protein degradation methods. To tackle this issue, microbial keratinases have emerged as promising tools for transforming resilient keratin materials into valuable products. We focus on the *Metalloprotease* (*MetPr*) gene isolated from novel *Pichia kudriavzevii* YK46, sequenced, and deposited in the NCBI GenBank database with the accession number OQ511281. The *MetPr* gene encodes a protein consisting of 557 amino acids and demonstrates a keratinase activity of 164.04 U/ml. The 3D structure of the protein was validated using Ramachandran's plot, revealing that 93% and 97.26% of the 557 residues were situated within the most favoured region for the *MetPr* proteins of template *Pichia kudriavzevii* strain 129 and *Pichia kudriavzevii* YK46, respectively. Computational analyses were employed to determine the binding affinities between the deduced protein and beta keratin. Molecular docking studies elucidated the optimal binding affinities between the *metalloprotease* (*MetPr*) and beta-keratin, yielding values of − 260.75 kcal/mol and − 257.02 kcal/mol for the template strains *Pichia kudriavzevii* strain 129 and *Pichia kudriavzevii* YK46, respectively. Subsequent molecular cloning and expression of the *MetPr* gene in *E. coli DH5α* led to a significantly higher keratinase activity of 281 ± 12.34 U/ml. These findings provide valuable insights into the potential of the *MetPr* gene and its encoded protein for keratin waste biotransformation, with implications for addressing environmental concerns related to keratinous waste accumulation.

## Introduction

With the world's population on the rise and an increasing demand for animal-based protein sources, livestock production has experienced significant growth worldwide. This expansion of livestock farming has resulted in the generation of substantial amounts of keratin-rich waste materials, including feathers from poultry, animal hairs, and horns^1^. Keratinous substances, constituting the third-largest category of natural polymers after cellulose and chitin, pose unique challenges due to their robust molecular structure^2^. They consist of tightly coiled polypeptide chains cross-linked by disulfide bonds, hydrogen bonds, salt bonds, and other cysteine-based bonds, rendering keratins resistant to degradation and insoluble in both water and organic solvents^3,4^.

Among these keratin-rich materials, feathers represent a significant by-product of the poultry industry. Globally, it is estimated that 8–10 million metric tonnes of feather waste are produced annually in the poultry sector, constituting approximately 40% of the total keratin biomass^5,6^ However, the management of feather waste remains a challenging task due to the inherent recalcitrance of keratin^7,8^. Existing methods for feather waste disposal primarily involve landfilling and incineration, both of which not only contribute to severe environmental pollution and the potential spread of pathogens but also result in the wastage of valuable renewable resources^9,10^.

Microbial biodegradation is the process by which microorganisms, such as bacteria and fungi, break down complex organic compounds into simpler, more readily usable forms. This ability of microorganisms to degrade various substances, including glycolipids, is a crucial aspect to consider in the context of environmental sustainability and waste management^11^. Algae and fungi play a role in bioremediation. Fungi, in particular, have the ability to degrade complex organic compounds, including PAHs. They have been used in various bioremediation applications, including the cleanup of contaminated soil and water^12^. One of the key abilities of microorganisms in biodegradation is the production of specific enzymes, such as laccases and peroxidases, which are involved in the degradation of different types of synthetic dyes^13^**.**

Keratinases represent a category of proteases capable of selectively breaking down and converting keratin into valuable protein resources. Most keratinases, categorised as serine or metalloproteases, demonstrate the ability to degrade fibrous and insoluble keratin materials with optimal temperatures ranging from 40 to 60 °C and pH values ranging from 7.5 to 9.0^14^. These keratinase-producing microorganisms are naturally found in various environments, particularly in locations rich in keratin, such as poultry waste, slaughterhouse residues, and tannery byproducts^15^. Thus far, microorganisms with the ability to produce keratinase have primarily included bacteria, actinomycetes, and fungi, among others^16^. However, native keratinase-producing microorganisms have limitations, including low enzyme yield and activity, which hinders their suitability for practical applications^17^. Due to microbes’ keratinolytic enzymes in the feed, pharmaceutical industries, leather, fertiliser, and biological control in agriculture^18^. As more keratinase genes have been identified, engineered expression systems have gained prominence. These systems, including *Escherichia coli*, *Bacillus *spp., and *Pichia pastoris*, are widely employed for the heterologous expression of keratinases^19^. To date, many keratinases have been isolated from keratin-degrading bacteria, fungi, and actinomycetes. For example, keratinase produced by *Bacillus licheniformis* has been intensively studied for its ability to degrade feather keratin, and its keratinase (*kerA*) gene has been sequenced and cloned^20^. Recently, using heterologous expression systems such as bacteria and yeast to produce keratinases has drawn considerable research interest due to the high yield and purity of proteins. However, only a few keratinase genes have been cloned and heterologously expressed^20–22^. The pGEM-T Easy-cloning system is the most powerful system for protein expression in *Escherichia coli*; the target gene is expressed under the control of strong T7 and SP6 promoter signals on the pGEM-T Easy-cloning vector.

In this work, we specifically focus on *MetPr* gene was isolated, sequenced, and subsequently deposited in the NCBI GenBank database. To gain a better understanding of the *MetPr* protein, we employed molecular modeling techniques to generate a 3D structure, which was further validated using Ramachandran's plot. The template proteins utilized for the modeling process were obtained from *Pichia kudriavzevii* strain 129 and *Pichia kudriavzevii* strain YK46. Additionally, molecular docking studies were conducted to investigate the protein's binding characteristics and provide valuable insights. The expression of the *MetPr* gene derived from *Pichia kudriavzevii* YK46 in *E. coli* DH5α, with the objective of enhancing keratinase activity.

## Material and methods

### Plasmids, reagents, media and strains

*Pichia kudriavzevii* strain YK46 NCBI Accession No. OK092586, which could efficiently degrade the feathers, was stored in our laboratory as described by Khalil et al.^23^. *E. coli DH5a* was grown at 37 °C for 12–18 h in Luria–Bertani (LB) broth or low-sault LB media. It contains (g/L): NaCl, 10.0; yeast extract, 5.0; and tryptone, 10.0. Yeast peptone glucose (YPG) agar medium (Himedia, West Chester, Pennsylvania, USA) was used for yeast growth. The basic medium used for isolation and fermentation of the feather-degrading microorganisms contained the following constituents (g/L): 0.2 M Na_2_HPO_4_, 0.1 M citric acid, 0.01% yeast extract, and 1% feather substrate, pH 7.2^24^. The pGEM-T Easy-cloning vector was obtained from (Promega Co., Madison, WI, USA) 3015 bp, Amp^r^, and total genomic DNA was extracted from *Pichia kudriavzevii* strain YK46 using a genomic DNA isolation kit (GeneDireX, Inc., Taoyuan, Taiwan). Plasmid DNA was extracted from *E. coli DH5a* by QIA prep spin miniprep kit. The T4 DNA ligase was obtained from NEB (USA) and TaKaRa (China).

### *Metalloprotease* (*MetPr*) encoding gene amplification

Firstly, the primers for the MetPr-encoding gene of *Pichia kudriavzevii* strain 129 were designed using its DNA sequence. Genomic DNA was extracted from the *Pichia kudriavzevii* YK46 strain (NCBI Accession No. OK092586)^23^ using a genomic DNA isolation kit and used as a template for amplifying the *MetPr* gene. Then, the *MetPr* encoding gene was amplified from *Pichia kudriavzevii* YK46 using specific primers as follows: forward primer *MetPr*-F (5′-ATGGCATTACAGATCTCTCCAAAGG-3′) and reverse primer *MetPr*-R (5′-TTATTCCTCCATAGTTTCCTCAGCAA-3′), derived from the *MetPr* gene sequence obtained using Primer3 software. This *MetPr* gene's 1674 nucleotide DNA sequence, encoding a protein of 557 amino acids, served as the template for polymerase chain reaction (PCR) amplification of the *MetPr* gene. All experimental procedures were carried out according to the manufacturer's instructions. The PCR reaction was performed using a GeneAmp PCR System 2400 thermal cycler (PerkinElmer Norwalk, Connecticut, USA) with 100 ng of genomic DNA in a 100 µl reaction containing master mix (TIANGEN, Beijing, China) and 5 µM primers^25^. The PCR programme consisted of 35 cycles, with initial denaturation at 95 °C for 5 min, followed by denaturation at 95 °C for 1 min, annealing at 55 °C for 1 min, and extension at 72 °C for 2 min per kilobase pair (kbp), with a final extension at 72 °C for 3 min. The PCR products were analysed on a 1% agarose gel, and then the 1674 bp band corresponding to the *MetPr gene was* extracted from the agarose gel using the FavorPrep GEL Purification kit (FAVORGEN, Biotech Corp., Ping Tung, Taiwan), purified using the Qiagen gel purification kit, and sent for sequencing.

### Bioinformatics analysis

With the help of the online translation tool ExPASY (http://web.expasy.org/translate), the *MetPr* protein sequence was obtained from the experimentally determined *MetPr* gene of *Pichia kudriavzevii YK46.* The deduced amino acids were analyzed using the NCBI protein blast (http://BLAST: (Basic Local Alignment Search Tool (nih.gov)). Blast searches the non-redundant protein sequence database, and hits with sequence identity were considered matches. Multiple Sequence Alignment (MSA) of protein sequences was carried out using the PRALINE online resource portal (http://www.ibi.vu.nl/programs/pralinewww/)^26,27^. Secondary structure prediction of the *MetPr* protein was carried out using the online NetSurfP-2.0 server^28^. Cluster analysis (phylogenetic tree) of *Pichia kudriavzevii* metalloprotease (MetPr) protein sequence was carried out using the MEGAX software (megax-software [ILRI Research Computing] (cgiar.org)).

### Structure prediction, validation and binding pockets prediction of *metalloprotease* (*MetPr*)

To predict the tertiary structure of *MetPr* protein, I-TASSER (http://I-TASSER server for protein structure and function prediction (zhanggroup.org) was used^29,30^. Five models were generated, and the best model with a confidence score (C-score, which is a measure of the quality of the predicted model) and potential energy was selected for further study^31,32^. The normal range of the C-score is − 5 to 2. Here, the C-score value and the structure quality are directly proportional. The best validation structure of *MetPr* protein was authenticated through the SAVES v6.0 (structure analysis and verification server version 6) online tool (http://SAVESv6.0—Structure Validation Server (ucla.edu)) and the ProSA server. SAVES v6.0 is a complete package of five programmes that test the overall consistency of a protein structure. Out of five programmes, we used VERIFY-3D to test the 3-D sequence profile for protein models and PROCHECK to validate structure through the Ramachandran plot^33,34^. Subsequently, a stereo image of the *MetPr* model illustrating the surface groove structure of the *MetPr* model was generated using “PyMol” software (DeLano Scientific LLC)^25^. The Site-Map module of the deepsite/playmolecule online tool (http://DeepSite: a binding pocket predictor using neural-networks [WEB APP] (playmolecule.com) was used for binding site predictions in the *MetPr* protein and ligand beta keratin^35^. For the identification of possible binding sites of amino acids, physical descriptors such as size, degree of enclosure or exposure, hydrophobic or philic character, tightness, and hydrogen-bonding possibilities were considered^36^.

### Protein–protein docking studies

The 3D structure of *MetPr* protein was processed using the MOE software are (Molecular Operating Environment (MOE) | MOEsaic | PSILO (chemcomp.com), eliminating water molecules, ions, and existing ligands. Hydrogen atoms were inserted into the receptor molecule using PyMOL (PyMOL | pymol.org). The substrate ligand, beta keratin (beta keratin, PubChem CID: 395651, PF02422), was prepared in pdb format using MOE and PyMOL. Docking studies were conducted using the HDock online tool (http://HDOCK Server (hust.edu.cn))^34,37^ to explore the binding mode between beta-keratin and the *MetPr* protein. The macromolecule file was saved in PDB format for docking. The 2-D hydrogen-bond interactions of the *MetPr* substrates were analysed using the 2D LigPlot programme (http://LigPlot+ homepage(ebi.ac.uk)), providing a graphical representation of hydrophobic and hydrogen bonds^25,31,36^.

### Molecular cloning and expression of *metalloprotease* (*MetPr*) in *E. coli DH5ɑ*

The amplified product *MetPr* of *P. kudriavzevii* YK46^23^ was inserted into the corresponding sites of the pGEM^®^-T Easy cloning vector using a ligation cloning kit (Promega Co., Madison, WI, USA). To obtain the recombinant plasmid through a ligation reaction using the enzyme T4 ligase Ligated DNA (20 µl) was added to 0.1 ml of freshly prepared competent cells with a transformation efficiency of 2.98 10^5^ cfu/lg DNA^38^. Recombinant plasmid was transformed into *E. coli DH5ɑ c*ells using the heat shock method, and the samples were incubated for 1 h at 37 °C. After incubation, samples were plated on LB agar plates containing 0.5 mM IPTG, 50 µg/mL ampicillin and 40 µg X-gal and incubated overnight at 37 °C. *MetPr* colonies were again streaked on an appropriate antibiotic-containing plate and incubated at 37 °C for 16–20 h. Positive colonies were monitored for extracellular expression at 37 °C by estimating *MetPr* activity^25,27,39,40^. Fifty milliliters Luria–Bertani (LB) broth medium containing ampicillin (50 μg/ml) was inoculated with overnight grown single colony of *E. coli* *DH5ɑ* that have been transformed with plasmid *pGEM-T-MetPr* and incubated at 37 °C with shaking for 16 h. grown culture was centrifuged at 4000 rpm for 10 min at 4 °C. Then, the supernatant was discarded, and cell pellets were used for recombinant plasmid isolation using the QIA prep spin miniprep kit, Germany, according to the manufacturer's protocol^27,41^. The transformed colonies containing the plasmid carrying the *MetPr* gene (*pGEM-T-MetPr*) were screened by colony PCR using *MetPr* gene-specific primers under the same PCR conditions used in *MetPr* gene amplification^27^.

### Preparation of crude enzyme extract and enzyme assay

Keratinolytic activity was assessed using soluble keratin (0.5%, w/v) as the substrate, which was prepared from white chicken feathers as described in reference Wawrzkiewicz et al.^42^. Native chicken feathers (10 g) were collected from different local poultry markets in Cairo, Egypt, treated with dimethyl sulfoxide (DMSO), heated, and precipitated with cold acetone. The resulting precipitate was washed, dried, and dissolved in a NaOH solution. The pH was adjusted, and the solution was diluted with Tris–HCl buffer (pH 8.0)^24^. To initiate keratinase production, a selected strain was transferred to a fermentation medium supplemented with 1% feather. The resulting supernatant, containing the enzyme, was collected for a quantitative keratinase assay. Keratinase enzyme activity was measured using keratin powder as a substrate. In brief, 1.0 ml of cell-free supernatant (crude enzyme) was incubated with 10 mg keratin powder in 1.5 ml phosphate buffer (pH 7.4) and incubated at 37 °C for 1 h as reported by Duarte et al.^24^. One unit (U/ml) of keratinolytic activity was defined as an increase in corrected absorbance of 280 nm (A280) with a control of 0.01 per minute under the conditions described above and calculated by the following equation:

$${\text{U}} = 4\, \times \,{\text{n}}\, \times \,{\text{A}}280/(0.01\, \times \,10),$$

where n is the dilution rate, 4 is the final reaction volume (ml), and 10 is the incubation time (min).

## Results

### *Metalloprotease* (MetPr) encoding gene amplification from novel *Pichia kudriavzevii YK46* and alignment in Genbank (Blast)

The designed primers specific to the *MetPr* gene were successfully at the first time amplified and subjected to sequencing. The resulting sequence was then compared to sequences available in the NCBI database using a BLAST search. Through this analysis, an open reading frame (ORF) spanning 1674 bp was identified, consistent with the expected length of the *MetPr* gene in novel *Pichia kudriavzevii* YK46. The encoded protein derived from this gene consists of 557 amino acids. The comparison also revealed a high degree of similarity, with a 99% match, between the *MetPr* gene from *Pichia kudriavzevii* YK46 and the *MetPr* gene of *Pichia kudriavzevii* strain 129. Consequently, the sequence obtained for the *MetPr* gene in *Pichia kudriavzevii* strain YK46 was deposited in the NCBI database under the accession number GenBank OQ511281. Following this, the nucleotide sequence of the *MetPr* gene was translated to its corresponding amino acid sequence, representing the protein under study. This amino acid sequence was aligned with three other *MetPr* sequences retrieved from the UniProt protein database. Furthermore, utilizing the InterProScan server from EMBL, it was determined that amino acid residues 1 to 557 belong to the *MetPr* family. The protein sequence of *MetPr* of *Pichia kudriavzevii* YK46 had 99% similarity with *MetPr* protein of *Pichia kudriavzevii* strain 129.

### Multiple sequence alignment (MSA) of *metalloprotease* (*MetPr*) protein

**Figure 1:**
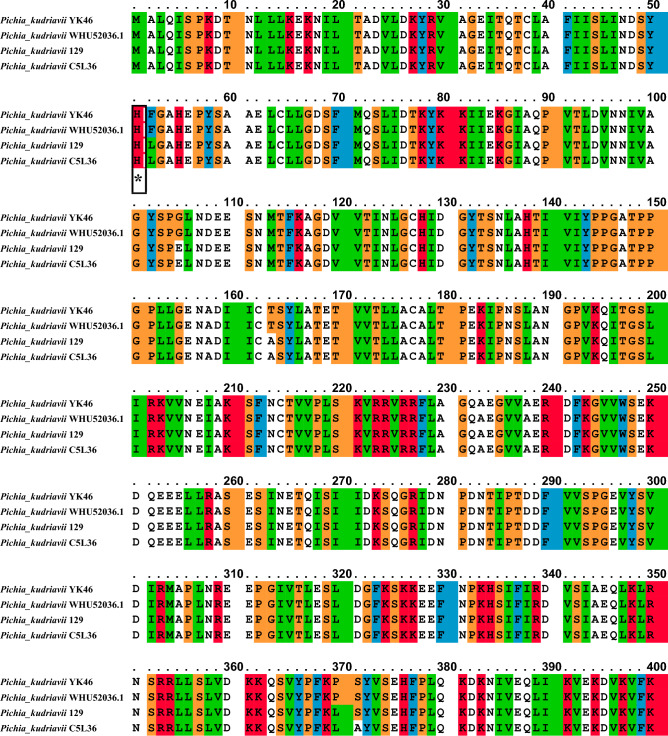

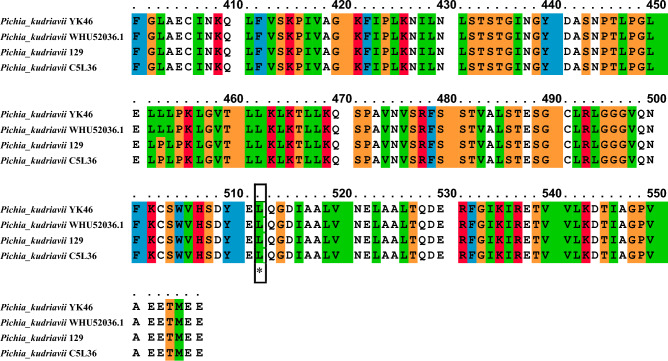
Multiple sequence alignment of the experimentally determined amino acids for *metalloprotease* (*MetPr*) amino acids from the *Pichia* family. Amino acid sequence alignment was performed by PSI-BLAST pre-profile processing (Homology-extended alignment) available from the PRALINE online resource portal (http://www.ibi.vu.nl/programs/pralinewww/). Active site residues across the metalloprotease are marked with a red ‘‘*’’.

During the multiple sequence alignment analysis of four sequences of *Pichia kudriavzevii* strain YK46, *Pichia kudriavzevii* strain 129, *Pichia kudriavzevii* WHU52036.1 and *Pichia kudriavzevii* C5L36_0E03930. Two consensus regions of the *MetPr* were identified. Among these sequences, the *MetPr* sequence of *Pichia kudriavzevii* YK46 exhibited the highest similarity to the *MetPr* sequence of *Pichia kudriavzevii* strain 129. All of these sequences belonged to the *Pichia* genus. Further investigation revealed that the active site of the *MetPr* in both *Pichia kudriavzevii* YK46 and the template strain *Pichia kudriavzevii* strain 129 was located at positions HIS51 and LEU512. Comparison of *MetPr* protein sequence of the *Pichia kudriavzevii* strain 129 and *Pichia kudriavzevii* strain YK46 strains, showed four substitutions of G105E, T163A, P370L and L453P in the* Pichia kudriavzevii* strain YK46 and *Pichia kudriavzevii* strain 129, as depicted in Fig. 1.

### Cluster analysis (phylogenetic tree) of *metalloprotease* (*MetPr*) protein

**Figure 2:**
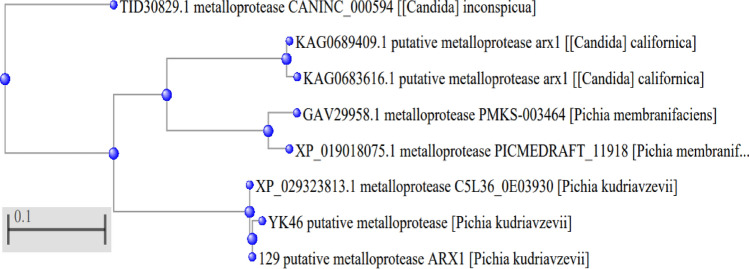
Phylogenetic tree of eight yeast strians *Pichia MetPr* protein sequences; constructed using the neighbor-joining method (MEGAX) software.

Following the sequence analysis, a phylogenetic study was conducted to determine the placement of the *MetPr* protein within the broader context of known *MetPr* family members. For this analysis, a curated dataset consisting of eight *MetPr* proteins from different organisms was selected from the UniProt protein database. One of these proteins included *MetPr* from *Pichia kudriavzevii* YK46. The comparative analysis revealed a high sequence similarity of 99% between the *MetPr* of *Pichia kudriavzevii* YK46 and the homologous *MetPr* proteins of *Pichia kudriavzevii* strain 129, both of which belonged to the *Pichia* genus. To construct the phylogenetic tree, the MEGAX software was utilised, using the *MetPr* sequences from the four *Pichia* strains. The results of the analysis showed the classification of *Pichia* genotypes into five primary clusters. Cluster one included TID 30829. 1 metalloprotease CANINC_000594 (*Candida inconspicua*) only. Cluster two comprised KAG0689409, 1 putative metalloprotease arx1 (Candida californica), and KAG0683616. 1 putative metalloprotease arx1 (Candida californica), Cluster 3 comprised GAV29958. 1 metalloprotease, PMKS-003464 (*Pichia membranifaciens*), and XP_019018075. 1 metalloprotease PICMEDRAFT_11918 (Pichia membranifaciens NRRL Y-2026), Cluster 4 comprised* XP_029323813. *1 metalloprotease C5L36_0E03930 (*Pichia kudriavzevii*) only, and Cluster five comprised the YK46 putative metalloprotease (*Pichia kudriavzevii*) and the 129 putative metalloprotease ARX1 (*Pichia kudriavzevii*), which were grouped together and closely related to the *MetPr*. Further details on the phylogenetic relationships among these strains can be seen in Fig. 2**.**

### Secondary structure prediction of *metalloprotease* (*MetPr*) protein

**Figure 3:**
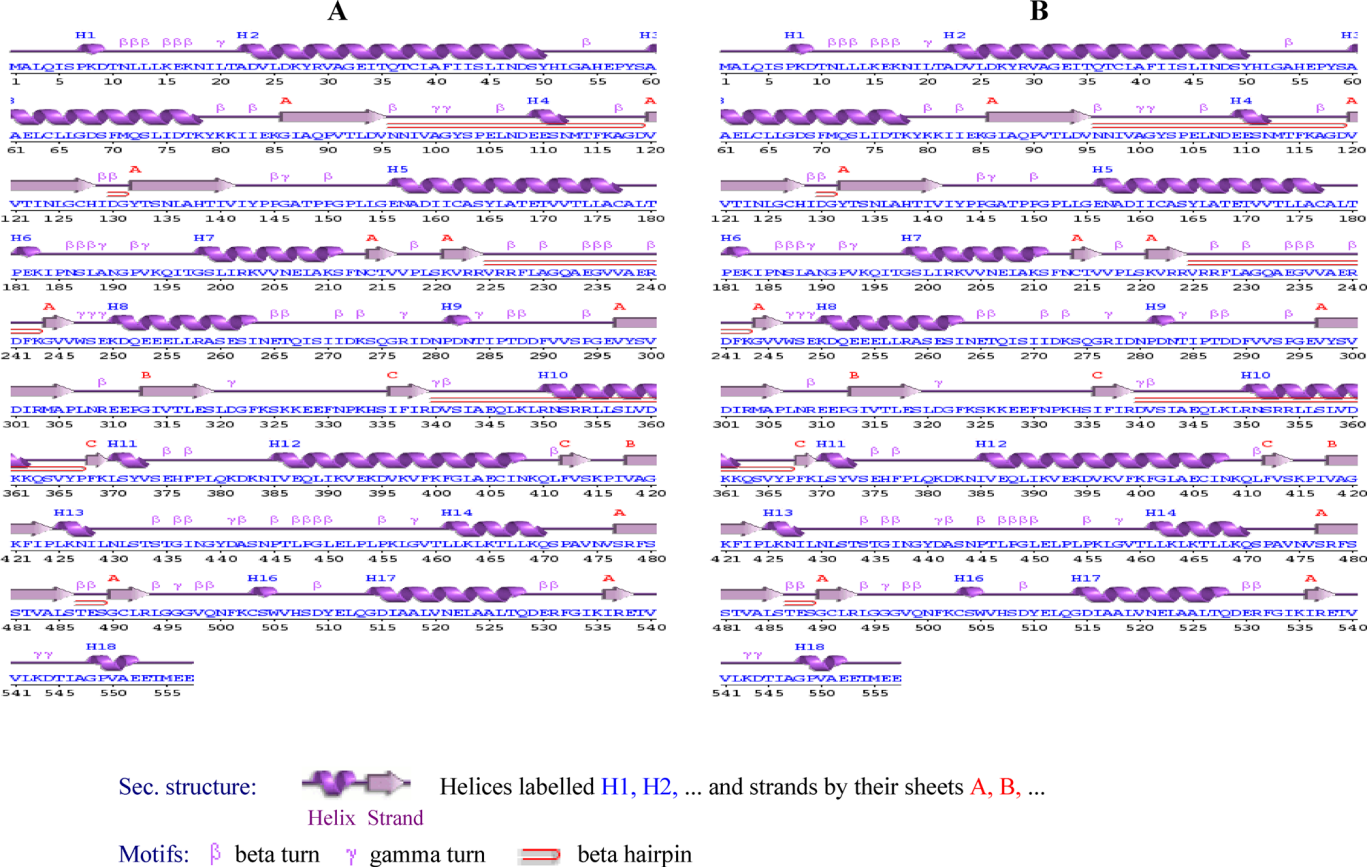
Predicted secondry structure of *MetPr*; (**A**) *Pichia kudriavzevii* strain 129. (**B**) *Pichia kudriavzevii* strain YK46*.*

The sequence alignment and secondary structure prediction of the *MetPr* protein from templates *Pichia kudriavzevii* strain 129 and *Pichia kudriavzevii* strain YK46 were conducted with the PDBsum server. According to the characterization of the *MetPr* model by the PDBsum, the predicted *MetPr* enzyme's topology and secondary structure are composed of 18 α-helices (H) and 57 β sheets (E) for *Pichia kudriavzevii* strain 129, and 18 α-helices (H) and 55 β sheets (E) for *Pichia kudriavzevii* strain YK46, respectively**,** as shown in Fig. 3.

### Structure prediction and validation of *Pichia kudriavzevii metalloprotease* (*MetPr*) protein

**Figure 4:**
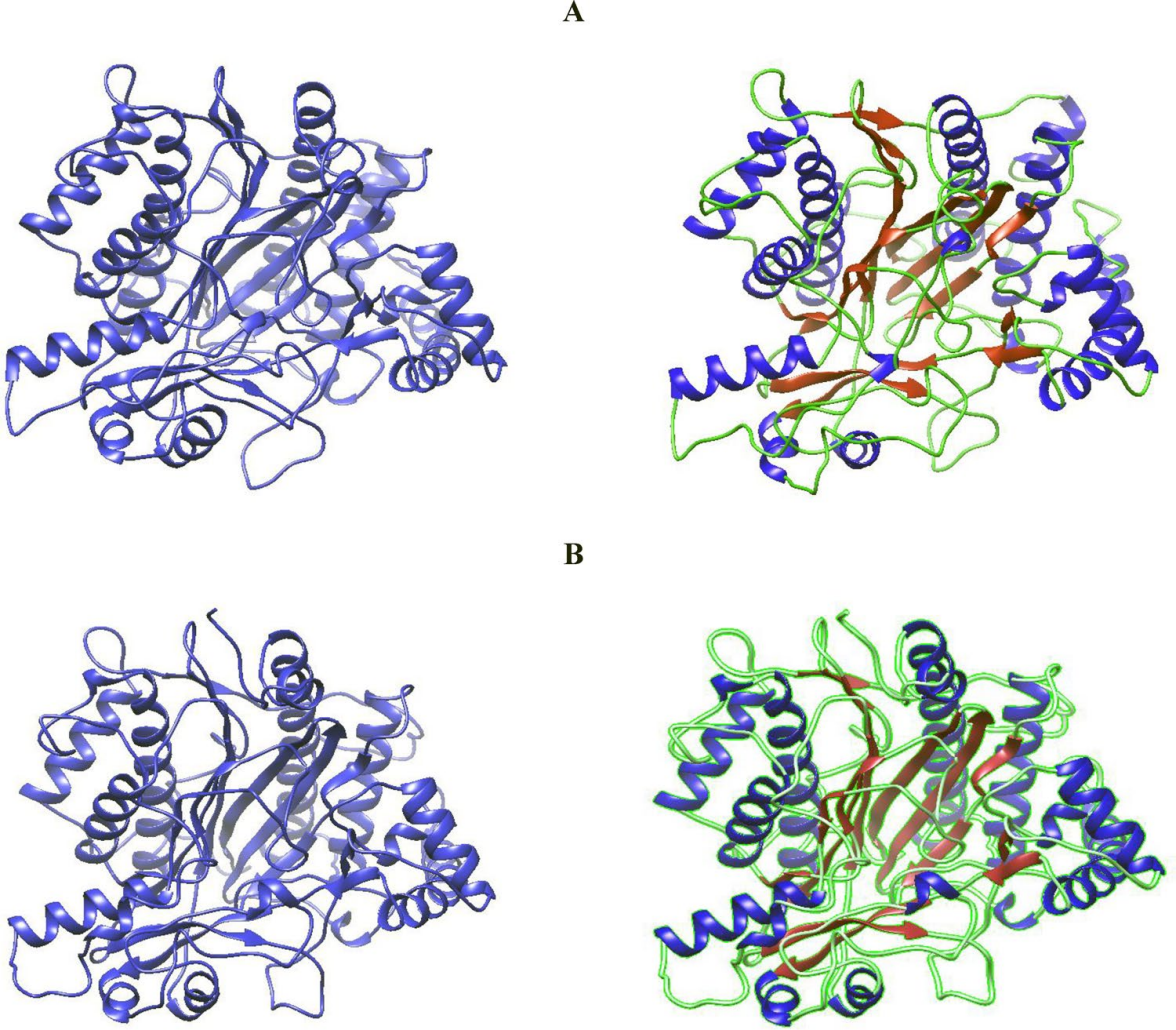
Predicted 3D modeled structure using a I-TASSER of; (**A**) template *Pichia kudriavzevii* strain 129, (**B**) strain *Pichia kudriavzevii* strain YK46 *MetPr*.

Five models were generated using I-TASSER for the 557-residue *MetPr* protein of the template *Pichia kudriavzevii* strain 129 and strain *Pichia kudriavzevii* YK46. Model 1 of both strains was selected based on the highest C-score, indicating a high confidence level. The estimated TM-score for the selected models was 0.46 ± 0.08, and the estimated RMSD values were 16.1 ± 2.7Å for the template strain *Pichia kudriavzevii* strain 129. The estimated TM-score for the selected models was 0.53 ± 0.24 and the estimated RMSD values were 14.4 ± 4.5Å for strain *Pichia kudriavzevii* YK46 (Fig. 4A and B)**.**

**Figure 5:**
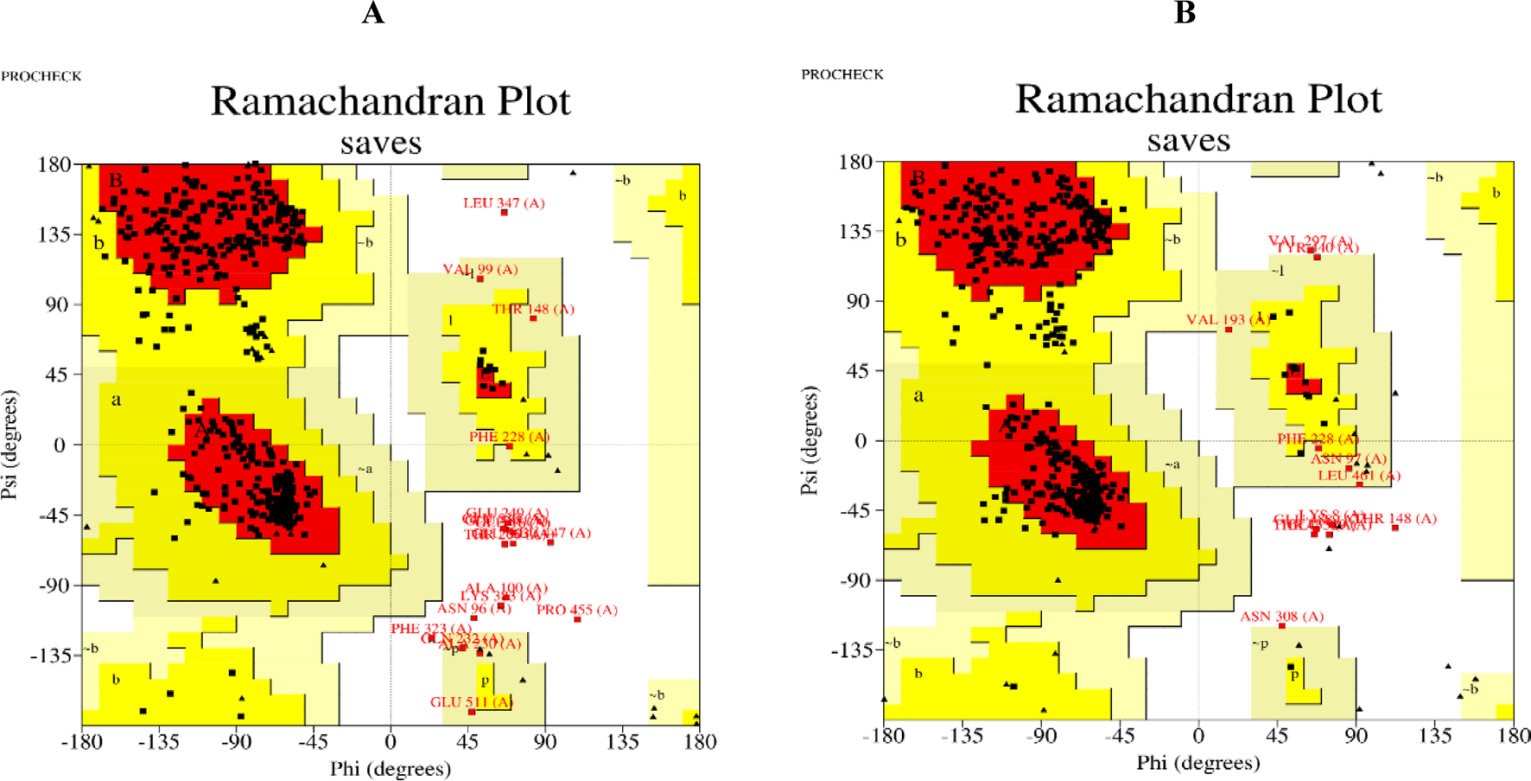
Structure validation: Ramachandran plot of; (**A**) template *Pichia kudriavzevii* strain 129, (**B**) *Pichia kudriavzevii* strain YK46 *MetPr*.

Final refined structure validation: The refined I-TASSER structure from the galaxy refine tool was verified using the SAVES v6.0 server. SAVES v6.0 consists of a package of five programs. Out of which, the result from the VERIFY-3D programme presented that 90.40 and 90.77% of the residues of *MetPr* protein were in the template strain *Pichia kudriavzevii* strain 129 and the template strain *Pichia kudriavzevii* strain 46, respectively. Had a 3D-1D arrangement, which is greater than the threshold value of 0.2. So, this model was successfully passed according to VERIFY-3D. ERRAT scores of 93% and 97.26% validated and predicted the overall sound quality of the structure in the template strain *Pichia kudriavzevii* strain 129 and the strain *Pichia kudriavzevii* YK46 *MetPr* protein, respectively, occurring in the most favored region, confirming the quality of the models (Fig. 5A and B). The PROCHECK analysis of the template strain *Pichia kudriavzevii* strain 129 *MetPr* protein showed that 87.4% of residues fell in the most favored regions of the Ramachandran plot, 8.9% in the additional allowed regions, 1.2% in the generously allowed regions, and 2.4% in the disallowed regions. Similarly, the PROCHECK analysis for strain *Pichia kudriavzevii* YK46 *MetPr* protein revealed that 87.4% of residues fell in the most favored regions, 10.2% in the additional allowed regions, 1.0% in the generously allowed regions, and 1.4% in the disallowed regions. The Ramachandran plot calculations indicated that the above data confirmed that the predicted model could be used for further experiments. which confirms that the overall model quality is good.

### *Metalloprotease* (*MetPr*) binding pocket prediction

The playmolecule/deepsite server was utilized to identify the active site amino acids. The active site analysis was performed on the template *Pichia kudriavzevii* strain 129 *MetPr* and strain *Pichia kudriavzevii* YK46 *MetPr*. Additionally, ligand beta-keratin was included in the analysis. For the template *Pichia kudriavzevii* strain 129 *MetPr*, the active site with a score of 0.993 was identified. It consists of one amino acid, HIS51, located at the centre of the active site, with an interface residue(s) of 1.201. In the case of ligand beta keratin, the active site analysis revealed a single amino acid, TYR77, at the active site centre, with an interface residue(s) of 1.201. For strain *Pichia kudriavzevii* YK46 *MetPr*, the active site with a score of 1.090 was identified. It consists of amino acid LEU512, located at the centre of the active site, with an interface residue(s) of 1.705. Similarly, for ligand beta keratin, the active site analysis showed a single amino acid, PRO54, at the active site centre, with an interface residue(s) of 1.705.

### Docking and molecular interaction studies

**Figure 6:**
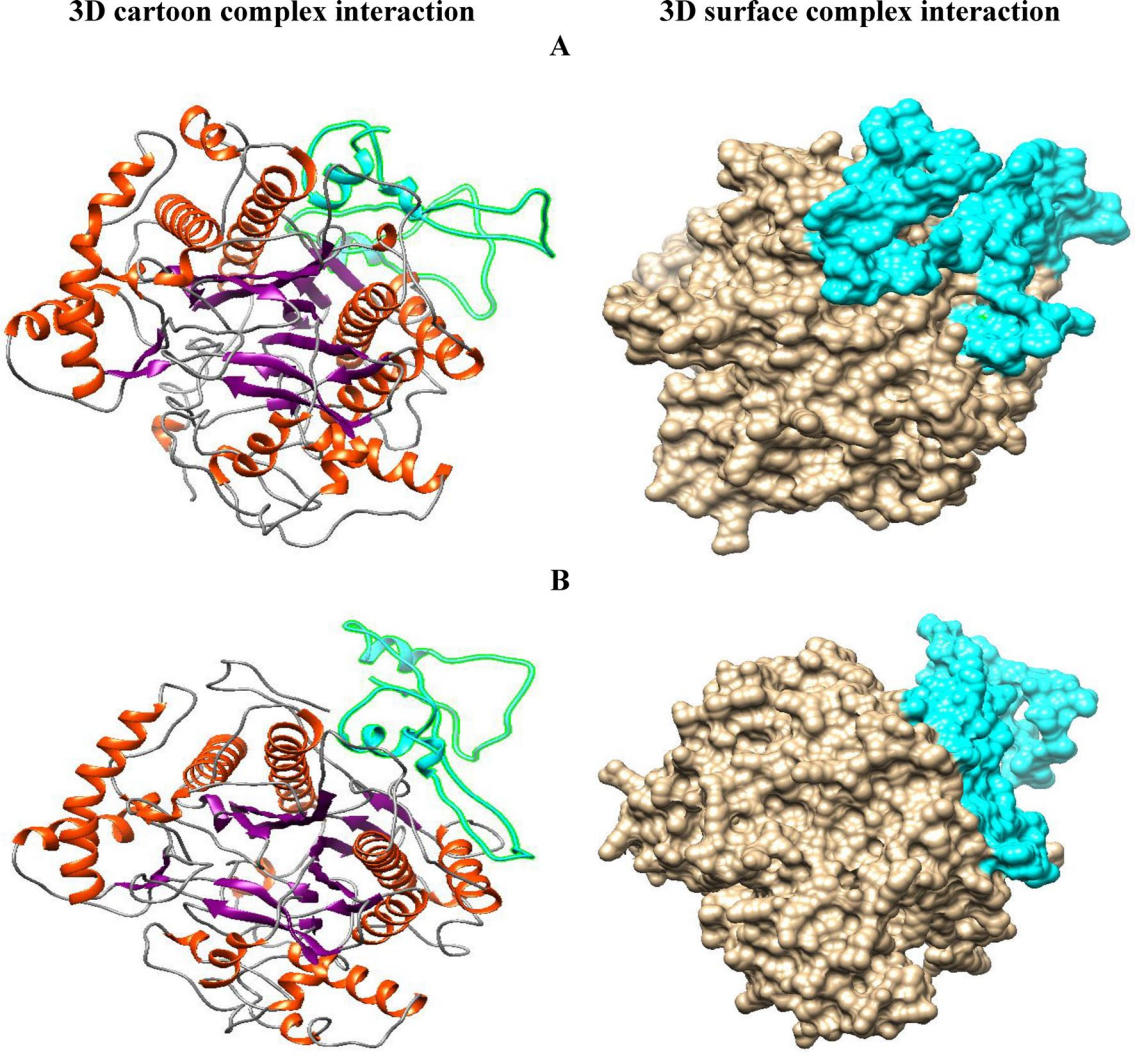
Chimera representation of protein–protein docking by HDock 3D cartoon complex interaction and 3D surface complex interaction of (**A**) template *Pichia kudriavzevii *strain 129, (**B**) *Pichia kudriavzevii *strain YK46; representation of molecular complex interaction of beta keratin (cyan color) with amino acids of the 3D model of *MetPr* protein receptor.

Docking studies were conducted using the HDock online tool to investigate the binding mode between the substrate beta-keratin and the 3D model of the *MetPr* protein. The docking results of beta-keratin with the 3D model of MetPr summarised the interactions. Specifically, the template *Pichia kudriavzevii* strain 129 *MetPr* exhibited an interaction with an affinity score of -260.75 kcal/mol, a confidence score of 0.9016, a ligand RMSD (Å) of 67.73, and involved the active site amino acids HIS51 (receptor) and TYR77 (ligand), with an interface residue(s) of 1.201, as depicted in Fig. 6A.

The docking analysis of *Pichia kudriavzevii* YK46 *metalloprotease* revealed an interaction with an affinity score of − 257.02 kcal/mol, a Confidence score of 0.8948, a Ligand RMSD (Å) of 53.03. The active site amino acids involved in this interaction were LEU512 (receptor) and PRO54 (ligand), with an interface residue(s) of 1.705, as illustrated in Fig. 6B.

### Cloning of *metalloprotease* (*MetPr*) encoding gene

**Figure 7:**
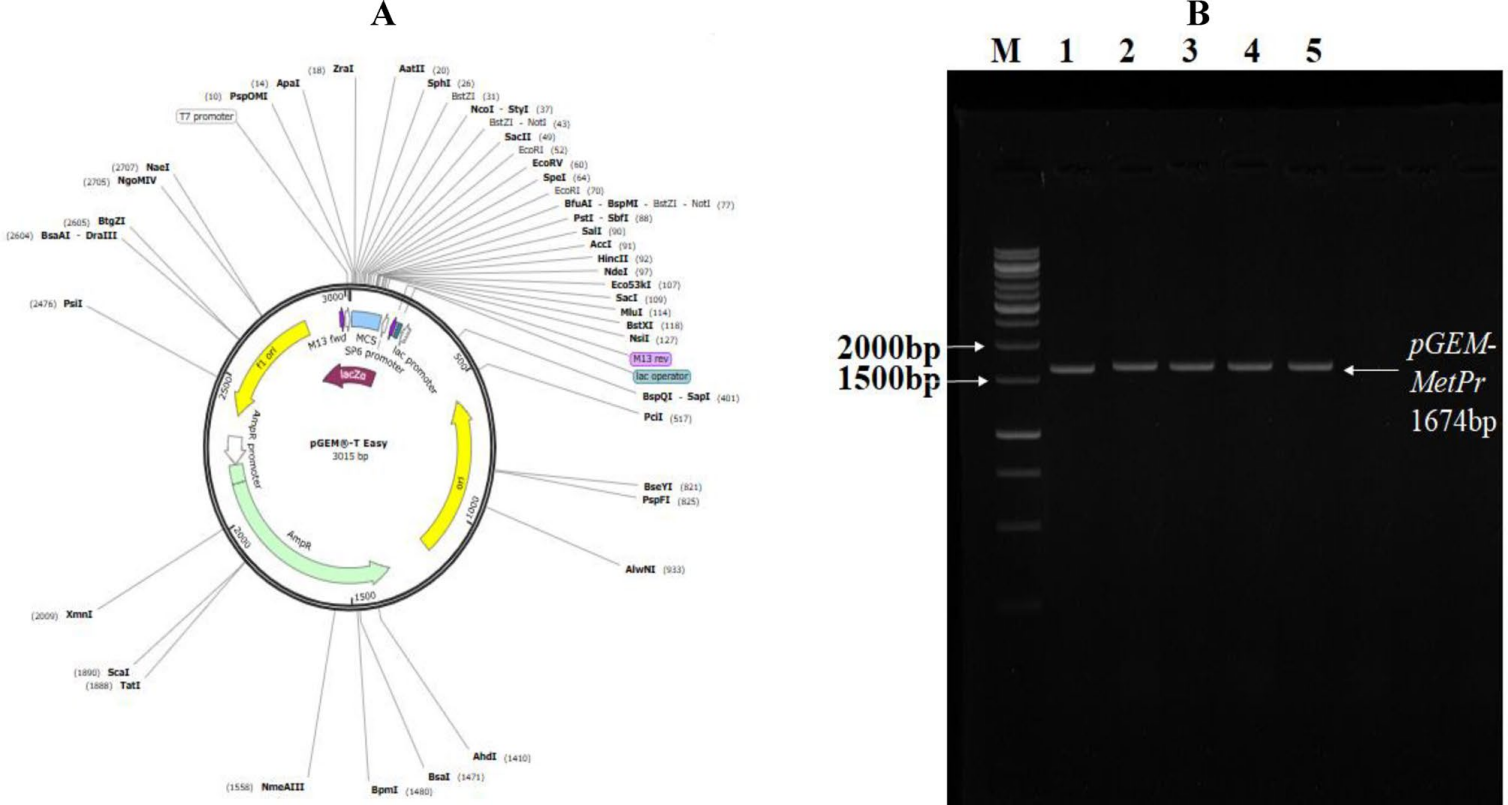
(**A**) pGEM-Teasy cloning vector, (**B**) Agarose gel electrophoresis of amplified PCR *MetPr* (1674 bp) of recombinant *pGEM- MetPr-* YK46; M, 10.000 bp DNA ladder (Invitrogen, California, USA).

The *MetPr* encoding gene, previously identified in *Pichia kudriavzevii* YK46 with a length of 1674 bp (GenBank: OQ511281), was successfully amplified using primers designed specifically for this gene. Gel electrophoresis was conducted to analyze the PCR products, resulting in the visualization of a 1.674 kb band, corresponding to the desired DNA amplicon, from the genomic DNA sample. The results indicated that an annealing temperature of 55°C was optimal for the amplification of the target 1674 bp DNA fragment. The 1674-bp DNA band was carefully excised from the agarose gel and purified. Subsequently, the purified DNA fragment was ligated into the pGEM^®^-T Easy Vector as illustrated in Fig. 7A using a ligation cloning kit, resulting in the generation of the recombinant plasmid named pGEM-T-*MetPr,* as illustrated in Fig. 7B. To introduce the recombinant plasmid into the expression host, *E. coli DH5α*, a heat-shock treatment was employed during transformation. The transformed *E. coli* cells were selected using ampicillin resistance and the white/blue screening method, which involved the use of IPTG and X-gal. Successful transformation was achieved, yielding various transformants of the *E. coli* strain. Plasmids were isolated from randomly selected *E. coli* transformants and analyzed through agarose gel electrophoresis. The analysis confirmed the presence of plasmid DNA bands in all tested *E. coli* transformants.The transformed colonies containing the plasmid carrying the *MetPr* gene (pGEM-*MetPr*) were selected by screened PCR using *MetPr* gene primers under the same PCR conditions used in *MetPr* gene amlification and subjected to agarose gel electrophoresis.

### Keratinolytic *metalloprotease* (*MetPr*) encoding gene expression

**Figure 8:**
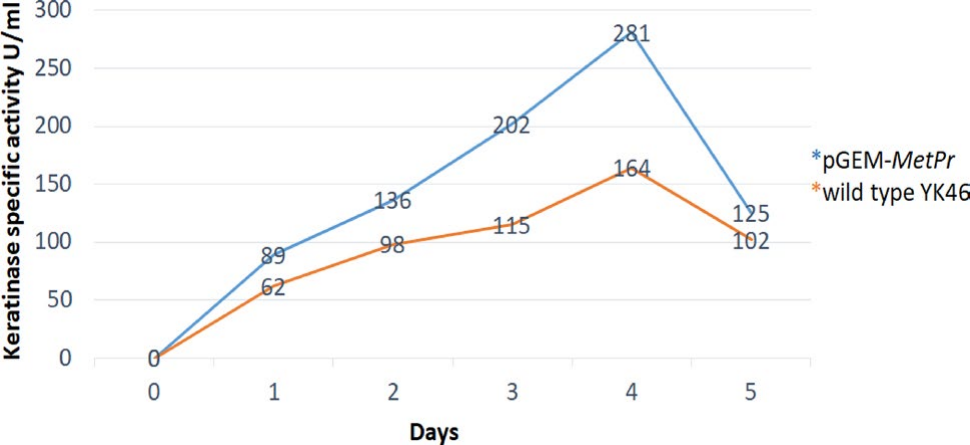
Keratinolytic *MetPr* specific activity of recombinant *E. coli DH5α* (pGEM-T- *MetPr*) and wild type YK46 at 0, 1, 2, 3, 4 and 5 days incubation.

Keratinolytic *MetPr* activity was evaluated using a fermentation medium supplemented with 1% feather as the sole carbon source. The experiment involved testing different strains: the recipient strains of *E*. *coli*, the donor strain *Pichia kudriavzevii* YK46, and two *E. coli* strains harboring the *MetPr* plasmid (*E. coli* (pGEM-T-*MetPr*). All cultures were incubated at 37°C with shaking at 120 rpm for a duration of 4 days. Samples were collected from each culture and centrifuged to obtain the supernatant, which served as the crude enzyme for the keratinolytic *MetPr* activity assay. The assay procedure followed previous protocols. The results revealed that the donor strain *Pichia kudriavzevii* YK46 and *E. coli* strains that acquired the *MetPr* plasmid (*E. coli* (pGEM-T-*MetPr*) displayed keratinolytic *MetPr* activity, whereas the *E. coli* recipient strains did not exhibit any activity. However. These findings provided confirmation of the functional activity of the cloned *MetPr* gene. The keratinolytic *MetPr* activity was consistently observed over the 4-day incubation period with feather substrate in both *E. coli* recombinant strains. On day 4, the highest activity was recorded, with approximately 281 ± 12.34 U/ml for *E. coli DH5α* (pGEM-T-*MetPr*), surpassing the activity of the wild-type donor strain *Pichia kudriavzevii* YK46, which exhibited an activity of 164.04 U/ml as shown in Fig. 8.

## Discussion

In a previous study conducted by Khalil et al.^23^, yeast *Pichia kudriavzevii* strain YK46 was obtained from different farmers in Cairo, Egypt. The researchers successfully in the first time amplified and sequenced the keratinolytic metalloprotease gene from novel *Pichia kudriavzevii* YK46 using designed primers. Through BLAST analysis, they identified an open reading frame (ORF) comprising 1674 bp, which codes for a protein consisting of 557 amino acids. Notably, the metalloprotease encoding gene and protein alignment from novel *Pichia kudriavzevii* YK46 exhibited a remarkable 99% similarity to the template *Pichia kudriavzevii* strain 129 *metalloprotease,* in a related study of Gupta et al.^25^ reported keratinase (*ker*) gene belongs to *Bacillus subtilis* RSE163 has 1342 bp nucleotides encoding 447 amino acids was successfully amplified by using gene specific primers. Nahar et al.^43^, *Bacillus licheniformis* MZK-05 produced an amplicon of 1,156 bp in a polymerase chain reaction while targeting the gene, *kerA*, responsible for the enzyme keratinase. Peng et al.^44^ reported the keratinase gene from *Bacillus licheniformis* BBE11-1 was successfully amplified by using gene specific primers. Rahimnahal et al.^45^ reported amplification of the keratinase gene from *Bacillus licheniformis* KRLr1 of 1050 bp and registered in the NCBI database under accession number MT482301.1^46^. Keratinase (KerS) gene was amplified from wild-type isolates (S1, S13, S15, S26, and S39) and their mutants (S1ems, S13uv, S13uv + ems, S15ems, S26uv, and S39ems). keratinase genes. Interestingly, keratinase KerS gene shared 95.5–100% identity to keratinase, thermitase alkaline serine protease, and thermophilic serine protease of the *B. cereus* group. Wang et al.^47^ reported the *kerT1* gene (1170 bp) was successfully amplified from feather-degrading strain *Thermoactinomyces s*p. YT06.

In our study, multiple sequence alignment investigations have revealed variations in the location of the active site among different strains of *Pichia kudriavzevii metalloprotease*. During the multiple sequence alignment analysis of four sequences of *Pichia kudriavzevii* strain, among these sequences, the *MetPr* sequence of *Pichia kudriavzevii* YK46 exhibited the highest similarity to the *MetPr* sequence of *Pichia kudriavzevii* strain 129. Further investigation revealed that the active site of the *MetPr* in both *Pichia kudriavzevii* YK46 and the template strain *Pichia kudriavzevii* strain 129 was located at positions HIS51 and LEU512. Comparison of *MetPr* protein sequence of the *Pichia kudriavzevii* strain 129 and *Pichia kudriavzevii* strain YK46 strains, showed four substitutions of G105E, T163A, P370L and L453P. Gupta et al.^25^ reported multiple sequence alignment of the experimentally determined *ker* enzyme with other subtilisin from the *Bacillus* family. The PDB codes of the other enzymes are listed. Nahar et al.^43^ reported Sequence alignment of the amino acids of mature protein sequence (274 aa) of *B. licheniformis* strains MZK-05 with the highly similar *KerA* and Subtilisin Carlsberg proteins of other strains. SC, Subtilisin Carlsberg. In the *KerA* from *B. licheniformis* MZK-05, discrepancies with other sequences, i.e., Tyr26 instead of Phe26, Asn86 and Ser211 instead of Ser86 and Asn211 respectively were observed. Peng et al.^48^ reported alignment of the pro‐peptide sequences of keratinases from different sources. Eight sequences corresponding to highly expressed and active keratinases were aligned for recombinant keratinase KerZ1 by 16%‐66% were obtained through the multiple sequence alignment of pro‐peptides. Rahimnahal et al.^45^ reported Protein sequence alignment of the keratinase Bacill*us licheniformis* KRLr1 and several closely related sequences. Almahasheer et al.^46^ reported multiple amino acid sequence analysis of *KerS* gene against S8 family peptidase Bacillus cereus group sequences retrieved from GenBank database. Abdel-Naby et al.^49^ reported multiple amino acid sequence analysis on *B. cereus* resulted in 19 amino acid substitutions that led to an improvement of the protease by about 31.17% compared with the wild type; nine of the amino acid substitutions improved the catalytic efficiency of the enzyme. In our study, to further analyze the relationships between these strains, a phylogenetic tree was constructed using MEGAX software, incorporating sequences from eight strains of *Pichia kudriavzevii metalloprotease* protein. The results demonstrated that *Pichia kudriavzevii* YK46 showed the highest similarity to template *Pichia kudriavzevii* strain 129, which were found to be closely related to *metalloprotease*. Rahimnahal et al.^45^ reported Circular phylogenetic tree analysis of amino acid sequences *KRLr1* and its similar sequences showed the highest similarity to *Bacillus licheniformis* keratinase that belongs to serine peptidase/subtilisin-like S8 family.The unrooted neighbor-joining (NJ) tree was constructed based on the alignment of the protein sequences with the high similarities.

In our study topology and secondary structure of the predicted *MetPr* protein from templates *Pichia kudriavzevii* strain 129 and *Pichia kudriavzevii* strain YK46 were conducted, revealed its units, which comprises 18 α-helices (H) and 57 β sheets (E) for *Pichia kudriavzevii* strain 129, and 18 α-helices (H) and 55 β sheets (E) for *Pichia kudriavzevii* strain YK46. The prediction of active sites in template *Pichia kudriavzevii* strain 129 and *Pichia kudriavzevii* YK46 revealed the presence of one active site each, with HIS51 and LEU512 at their centers, respectively. Rahimnahal et al.^45^ reported the NetSurfP-2.0 server showed that the *KRLr1* consists of a secondary structure containing 25% α-helix and a 27% β-sheet. The amino acids predicted for the active site cleft of *KRLr1* are located close to the structure of *Bacillus subtilis* subtilisin E. These amino acids as follows: His75, Asp136, Asn138, His140, Leu172, Ser175, Ser177, Ser232, Ala279, Thr292, Ans294, and Ser297 for *KRLr1*which are shown in orange sticks. Using the superimposition command in Chimera V13.1, the modeled structure of the *KRLr1* was compared with three high-similar structure relative to the *Bacillus subtilis* subtilisin E. Protein 3D structure prediction software plays a crucial role in modeling protein sequences that lack structural information. It aids in understanding the interaction between proteins and ligands or other molecules, thereby enhancing our comprehension of the relationship between protein sequence, dynamics, structure, and function^28^. In our study, To perform homology modeling of the *metalloprotease* proteins from template *Pichia kudriavzevii* strain 129 and *Pichia kudriavzevii* YK46, I-TASSER, ROSETTA and Phyre2 were used^29,30^. Lastly, for further experimentation, the best model predicted by I-TASSER was chosen which is an interactive framework designed for template-based predictions of protein structure and function. For multiple threading alignments and iterative structural assembly simulations, I-TASSER first generates full-length atomic structural models using the highest significant template, followed by atomic level structure refinement starting from the target protein amino acid sequence^50,51^. I-TASSER uses LOMETS^52^, five models were generated and the best model with a confidence score (C-score which is a measure of quality of the predicted model) and potential energy was selected for further study^53^ and^32^. The calculated C-score was based on the consequence of alignment among threaded structures, used template and the parameters generated during structure assembly simulations^34,52^. The normal range of C-score is − 5 to 2. Here the C-score value and the structure quality are directly proportional. Among these models, the one with the highest C-score was selected for further analysis. A high C-score indicates a model with greater confidence and reliability^28^. Five models were generated using I-TASSER for the 557-residue *MetPr* protein of the template *Pichia kudriavzevii* strain 129 and strain *Pichia kudriavzevii* YK46. Model 1 of both strains was selected based on the highest C-score, indicating a high confidence level. The estimated TM-score for the selected models was 0.46 ± 0.08, and the estimated RMSD values were 16.1 ± 2.7Å for the template strain *Pichia kudriavzevii* strain 129. The estimated TM-score for the selected models was 0.53 ± 0.24 and the estimated RMSD values were 14.4 ± 4.5Å for strain *Pichia kudriavzevii* YK46.

Validation of *MetPr* using SAVES v6.0 is a complete package of five programs that test the overall consistency of a protein structure. Out of five programs, we used VERIFY-3D to test the 3-D sequence profile for protein models and PROCHECK to validate structure through the Ramachandran plot. PROCHECK checks the stereo chemical nature of a protein structure by analyzing residue-by-residue geometry and overall structure geometry^33,34^. In our study, The modeled structure3D was validated using the Ramachandran plot calculations demonstrated that the majority of the 557 residues in both template *Pichia kudriavzevii* strain 129 and *Pichia kudriavzevii* YK46 were located in the most favored region. Specifically, 93% and 97.26% of the total residues for template *Pichia kudriavzevii* strain 129 and Pichia kudriavzevii YK46, respectively, fell into in most favored region. This finding indicates the stability and reliability of the Ramachandran plot, as well as the results obtained from ERRAT, VERIFY3D, and PROSA models, which are valuable tools in facilitating in silico studies^31^. Gupta et al.^25^ reported *Bacillus subtilis* RSE163 keratinase ker gene representing 1342 bp nucleotides encoding 447 amino acids. The modeled structure3D was validated using ramachandran’s plot showing 305 residues (84.3%) in most favoured region. Topology and secondary structure of the predicted keratinase enzyme revealed its units, which comprises of sixteen stranded beta sheets (AE) and twelve alpha helices (H1–H12), whereas motifs represented as beta (β) and gamma (Y) turn along with beta hairpin (Ɔ).

The mature peptide consists of 29, 76, and 274 amino acids, respectively. To predict the protein structure of the keratinase *KerZ1*, homology modeling was performed using the Swiss-Model tool. The analysis revealed that the pro-peptide portion of the protein contained two α-helices and five β-sheets^54^. α-helices and β-sheets are generally recognized as crucial elements influencing protein structure and function^55^. Therefore, conducted sequential truncations of these structural components to investigate the importance of the pro-peptide in the synthesis of the mature enzyme.

Patni et al.^34^, conducted tertiary structure modeling: I-TASSER predicted the tertiary structure of the protein. The C score of − 0.68 with a TM-score of 0.63 ± 0.14 and RMSD of 8.4 ± 4.5 Å was chosen for the further experiment TM-score greater than 0.5 suggests a valid topology model, and a TM-score less than 0.17 implies random similarity. And conducted structure validation: The refined I-TASSER structure from the galaxy refine tool was verified using the SAVES v5.0 server. Through PROCHECK Ramachandran plot was evaluated. Ramachandran plot depicted that 82.7% of protein residues were in the most favored region. Furthermore, 13.0% and 1.7% residues were found in allowed and generously allowed areas, respectively, and only 2.7% of the residues were present in the disallowed region. Nahar et al.^43^ reported the three dimensional models of *KerA Bacillus licheniformis* MZK-05MZK05 were built with ProMod Version 3.70 simulating the 3-D models of PDB templates 4gi3.1.A, 1yu6.1.A, 1c3l.1.A whose sequence identities with our protein sequence were 98.54%, 97.81% and 98.18% respectively. Models simulating 4gi3.1.A, 1yu6.1.A and 1c3l.1.A were named Model 01, Model 02 and Model 03 respectively. QMEAN scores estimated for Model 01, 02 and 03 were 0.60, 0.11 and -0.08 and were predicted with none, 1 × Ca2 + and 2 × Ca2 + ligand binding sites respectively. Rahimnahal et al.^45^ reported *Bacillus subtilis* keratinase KRLr1 PDB was modeled by MODELLER V.9 with assuming of 3WHI PDB as a template structure. KRLr1 PDB structure was refined by the ModRefine server and validation of the modeled KRLr1 was surveyed in PROSA and PROCHECK servers. The Verify-3D scores and ERRAT quality factors were estimated at 58 and 93.06%, respectively. Based on Ramachandran graph results, 90% of the amino acids were in the favored, 8% in the allowed, and 2% in the outlier regions after refinement which indicates that the amino acids *KRLr1* are well-positioned at the angles phi (φ) and psi (ψ). The validation results show that KRLr1 has been properly modeled. Almahasheer et al.^46^ reported model validation of keratinase 3D structure by Ramachandran plot showing 91.98% favored region and 1.43% of the disallowed region. Amino acids not in the favored region are A324 SER, A46 GLN, A160 VAL, A137 ASP, and A114 PRO.

Molecular docking is a widely used technique for studying drug-receptor interactions and predicting the binding affinity of small molecules to their target proteins^56^. In our molecular docking studies of *metalloproteases*, beta keratin exhibited optimal binding affinities of -260.75 and -257.02 kcal/mol for template *Pichia kudriavzevii* strain 129 and *Pichia kudriavzevii* YK46, respectively. Docking studies have also been utilized to enhance the catalytic efficiency of keratinases from *Bacillus licheniformis* and *Stenotrophomonas* sp.^36,57^. In another study by Gupta et al.^25^, the cloned keratinase-encoding gene of *Bacillus subtilis* RSE163 docking studies using extra precision (XP) method of Glide showed optimum binding affinities with the drugs Acitretin (− 39.62 kcal/mol), Clobetasol propionate (− 37.90 kcal/mol), Fluticasone (− 38.53 kcal/mol), Desonide (− 32.23 kcal/mol), Anthralin (− 38.04 kcal/mol), Calcipotreine (− 21.55 kcal/mol) and Mometasone (− 28.40 kcal/mol) in comparison to other psoriasis drugs. In another investigation conducted by Banerjee et al.^36^, molecular docking studies were performed using phenylmethylsulfonyl fluoride (PMSF) to predict the active site of *Bacillus licheniformis* alkaline serine protease, which exhibited 100% sequence similarity with the selected *Bacillus* genus sequence structure. Ten docking sites were identified, and two of them were predicted and selected as active sites for keratinase belonging to the *Bacillus* genus. Fang et al.^57^ reported docking of *Stenotrophomonas sp*. keratinase has been reported in order to enhance their catalytic efficiency. Patni et al.^34^, conducted protein–protein docking: molecular docking was performed, the interaction of PE_PGRS39 with integrin’s and SH3 domain was modeled by pyDock. performed docking with three integrin’s with PDB code: 4m76 (β2 integrin; score: − 129.39), 4o02 (β3 integrin; score: − 122.25) and 3vi3 (α5β1), out of that α5β1 showed maximum affinity (total binding energy) of − 138.678. Rahimnahal et al.^45^ reported docking of KRLr1-FK4 and KRLr1-FK12 structures were then introduced to the HADDOCK server for docking. The HADDOCK statistical results of KRLr1-FK4 and KRLr1- FK12 complexes were compared. The HADDOCK Scores with the highest negative binding energy from clusters relative to the KRLr1- FK4 and KRLr1-FK12 complexes were selected. Overall HADDOCK results showed that LRRk1 could interact with FK4 (− 130.7 ± 1.5 kcal/ mol) with higher negative energy than FK12 (− 111.1 ± 22 kcal/mol). Almahasheer et al.^46^ revealed docking studies revealed the impact of substitutions on the superimposed structure and an increase in binding of mutant D137N of KerS13uv + ems (affinity: − 7.17; S score: − 6.54 kcal/mol) and seven mutants of KerS26uv (affinity: − 7.43; S score: − 7.17 kcal/mol) compared to the wild predicted structure (affinity: − 6.57; S score: − 6.68 kcal/mol).

Currently, most molecular studies and characterizations of keratinases emphasized on isolating the keratinases from various microorganisms and transforming them into *E. coli* hosts. As *E. coli* is the most studied model microorganism, its usage as an expression host for recombinant keratinase is extensive. Expressing keratinase in *E. coli* conferred several advantages compared to the native host, namely, (1) various strains of *E. coli* could be used to optimize the production of recombinant keratinase; (2) myriads of vectors could be used to control the induction of recombinant keratinase and to simplify the purification processes;(3) the expression of recombinant keratinases could be adjusted by constitutive, inductive, or auto-inducible promoters; and (4) the cloned recombinant keratinase could be subjected to rational design to further intensify its characteristics^17^. The genetic flexibility of *E. coli* and the wide selection of expression vectors available make *E. coli* the most used heterologous host for the expression of recombinant keratinase. Most of the molecular studies of recombinant keratinases used the pET vector for keratinase expression in *E. coli*^25^. The presence of the ColE1 origin, lacl operon, as well as the T7 transcription and translation signals, makes the pET vector suitable for protein expression in *E. coli*. It seems that the T7 promoter confers viable expression in E. coli, as shown by numerous investigations that utilized the pET plasmid containing the T7 promoter. Additionally, the tac promoter was reported to facilitate the efficient expression of the recombinant keratinase in *E. coli*^58^. The usage of pET vectors allows the recombinant keratinase to be expressed as tagged protein, in which the keratinase is fused with His-tag on its C-terminal. The presence of His-tag allows the recombinant keratinase to be purified using Ni^2+^ affinity chromatography without suffering from misfolding incidences^47^.

Subsequently, in our study researchers successfully cloned and expressed the keratinolytic *metalloprotease* gene from *Pichia kudriavzevii* YK46 using the pGEM-Teasy cloning vector under T7 and SP6 strong promotors. The positive clones obtained from the experiment were examined to assess the extracellular keratinase expression at 37 °C. The results showed a significantly higher activity of keratinase in the transformed pGEM-*MetPr*-YK46 strain, which recorded an activity of 281 ± 12.34 U/ml, compared to the wild-type strain *Pichia kudriavzevii* YK46, which recorded an activity of 164.04 U/ml. This indicates that the transformation with the pGEM-*MetPr*-YK46 construct led to an increase in keratinase activity.

There are several investigations that involved cloning and expression of keratinases in which keratinases from various native hosts such as *Bacillus spp*., *Streptomyces spp*., *P*. *aeruginosa, A. viridilutea, Fervidobacterium *sp., and *Thermophilus* sp. have been cloned and subsequently expressed in heterologous hosts such as *E. coli, Bacillus subtilis* and* Pichia*. *pastoris* to enhance the production of recombinant keratinases or to be subjected to further augmentation^59,60^**.** However, the production of recombinant keratinases is still insufficient for large-scale demands^14^**.** In a study by Abd El-Aziz et al.^61^ sequential mutagenesis applied to *Streptomyces werraensis* KN23 using UV, H_2_O_2_, and SA, resulting in several mutants. The best keratinolytic efficiency mutant was designated as SA-27 and exhibited a keratinase activity of 106.92 U/ml. In a study by Nahar et al.^43^, found that *Bacillus licheniformis* MZK-05 produced a 1156 bp amplicon, targeting the kerA gene responsible for keratinase enzyme production. They then cloned this amplicon into the plasmid vector pGEX-6p-2 to express it in *Escherichia coli* BL21. This expression led to a significant increase in keratinolytic (196 U/mL) activities, indicating improved functionality of the keratinase. Gupta et al.^25^ demonstrated the expression of a cloned *Bacillus subtilis* RSE163 keratinase gene. The *ker* gene expressed in *E. coli* exhibited significantly higher keratinase activity, with an activity of 450 ± 10.43 U. In, Peng et al.^44^ achieved successful expression of the keratinase gene from *Bacillus licheniformis* BBE11-1 in *Bacillus subtilis* WB600. They accomplished this by substituting the promoter and screening ribosome-binding sites, resulting in an improved recombinant keratinase KerZ1 with an activity level of 45.14 KU/ml. Rahimnahal et al.^45^ conducted a study in which they successfully expressed the Keratinase gene intracellularly within *Escherichia coli* BL21(DE3) using the pET-21b ( +) vector.. Expressed KRLr1 was purified and a yield of 85.96% and then refolded. Further, Su et al.^62^ employed a recombinant mutant M7 of *B. subtilis* keratinase to obtain 3040 U/mL of extracellular keratinase by continuous fermentation for 32 h in a 15-L bioreactor. Wang et al.^47^ The kerT1 gene (1170 bp) encoding keratinase from feather-degrading strain *Thermoactinomyces* sp. YT06was cloned and expressed in *Escherichia coli* BL21(DE3). Purifed recombinant keratinase (rKERTYT) was achieved at a yield of 39.16% and 65.27-fold purifcation with a specifc activity of 1325 U/mg. Mwanza et al.^63^, reported primers were designed on the selected gene of interest, which was amplified from the genome of *Chryseobacteriumcarnipullorum*. The gene coding for peptidase M64 was further cloned, propagated and expressed in *E. coli* BL21 [DE3] cells. Liao et al.^64^ reported keratinase from *Bacillus amyloliquefaciens* K11 successfully expressed of expression elements, including signal peptides and promoters, were optimized, and the combination of signal peptide SPSacC with promoter Pdual3 owned the best performance, keratinase activity aggrandized by 6.21-fold.

## Conclusion

This study focused on the potential of the Metalloprotease (*MetPr*) gene from *Pichia kudriavzevii* YK46 in the biotransformation of keratin waste. The *MetPr* gene was successfully sequenced and deposited in the NCBI GenBank database. The protein encoded by the gene exhibited a high keratinase activity, indicating its potential for degrading keratin-rich waste materials. Computational analyses and molecular docking studies further supported the binding affinity between *MetPr* and beta keratin. Additionally, molecular cloning and expression of the *MetPr* gene in *E. coli DH5α* led to an increased keratinase activity. These findings highlight the potential use of microbial keratinases, specifically *MetPr*, in managing keratin waste. Future research in this field can focus on further characterizing the *MetPr* protein and its enzymatic properties. This can include studying its substrate specificity, pH and temperature optima, and stability under different conditions. Additionally, the optimization of the expression system for *MetPr* can be explored to improve the yield and scalability of production. Further studies can also investigate the potential application of *MetPr* in other industries, such as textile, leather, and cosmetics, where keratin waste is generated.

## Data Availability

The sequenced identification of *Pichia kudriavzevii YK46* has been deposited in the NCBI database under accession numbers OK092586. The sequenced metalloprotease gene has been deposited in the NCBI database under accession number OQ511281. All the remaining data supporting the findings of this study are available within the article.

## Abbreviations

*MetPr*: *Metalloprotease*
*P. kudriavzevii*: *Pichia kudriavzevii*
LB: Luria–Bertani medium
YPG: Yeast peptone glucose medium
DMSO: Dimethyl sulfoxide
PCR: Polymerase chain reaction
NCBI: National Center for Biotechnology Information
C-Score: Confidence score
MOE: Molecular operating environment
Amp: Ampicillin
BLAST: Basic local alignment search tool
ORF: Open reading frame
MSA: Multiple sequence alignment
EMBL: European Molecular Biology Laboratory
MEGAX: Molecular evolutionary genetics analysis
I-TASSER: Iterative threading assembly refinement
TM-score: Template modeling score
RMSD: Root mean square deviation
IPTG: Isopropyl β-d-1-thiogalactopyranoside
X-Gal: 5-Bromo-4-chloro-3-indolyl-β-d-galactopyranoside
